# *CCND1* mutations increase protein stability and promote ibrutinib resistance in mantle cell lymphoma

**DOI:** 10.18632/oncotarget.12434

**Published:** 2016-10-04

**Authors:** Atish Mohanty, Natalie Sandoval, Manasi Das, Raju Pillai, Lu Chen, Robert W. Chen, Hesham M. Amin, Michael Wang, Guido Marcucci, Dennis D. Weisenburger, Steven T. Rosen, Lan V. Pham, Vu N. Ngo

**Affiliations:** ^1^ Division of Hematopoietic Stem Cell and Leukemia Research, Gehr Leukemia Center, Beckman Research Institute of City of Hope, Duarte, CA 91010, USA; ^2^ Toni Stephenson Lymphoma Center, Beckman Research Institute of City of Hope, Duarte, CA 91010, USA; ^3^ Department of Pathology, Beckman Research Institute of City of Hope, Duarte, CA 91010, USA; ^4^ Department of Information Sciences, Beckman Research Institute of City of Hope, Duarte, CA 91010, USA; ^5^ Department of Hematology and Hematopoietic Cell Transplantation, Comprehensive Cancer Center, Beckman Research Institute of City of Hope, Duarte, CA 91010, USA; ^6^ Department of Hematopathology, The University of Texas MD Anderson Cancer Center, Houston, TX 77030, USA; ^7^ Department of Lymphoma/Myeloma, The University of Texas MD Anderson Cancer Center, Houston, TX 77030, USA

**Keywords:** cyclin D1, somatic mutations, ibrutinib resistance, mantle cell lymphoma

## Abstract

Mantle cell lymphoma (MCL) is characterized by the t(11;14) translocation, which leads to deregulated expression of the cell cycle regulatory protein cyclin D1 (CCND1). Genomic studies of MCL have also identified recurrent mutations in the coding region of *CCND1*. However, the functional consequence of these mutations is not known. Here, we showed that, compared to wild type (WT), single E36K, Y44D or C47S *CCND1* mutations increased CCND1 protein levels in MCL cell lines. Mechanistically, these mutations stabilized CCND1 protein through attenuation of threonine-286 phosphorylation, which is important for proteolysis through the ubiquitin-proteasome pathway. In addition, the mutant proteins preferentially localized to the nucleus. Interestingly, forced expression of WT or mutant CCND1 increased resistance of MCL cell lines to ibrutinib, an FDA-approved Bruton tyrosine kinase inhibitor for MCL treatment. The Y44D mutant sustained the resistance to ibrutinib even at supraphysiologic concentrations (5–10 μM). Furthermore, primary MCL tumors with *CCND1* mutations also expressed stable CCND1 protein and were resistant to ibrutinib. These findings uncover a new mechanism that is critical for the regulation of CCND1 protein levels, and is directly relevant to primary ibrutinib resistance in MCL.

## INTRODUCTION

CCND1 is an important cell cycle regulator in many cell types. CCND1 binds and activates cyclin-dependent kinase (CDK) 4, which in turn phosphorylates and inactivates retinoblastoma (Rb) protein, leading to G1/S cell cycle progression [1, 2]. CCND1 accumulates in the nucleus during the G1 phase and translocates to the cytoplasm for degradation by the ubiquitin-proteasome system when cells enter the S phase [3, 4]. Cell cycle-dependent proteolysis of CCND1 requires phosphorylation of threonine-286 (T286) residue, which is predominantly mediated by glycogen synthase kinase 3 beta (GSK3B) [5]. Other kinases including DYRK1B and p38 MAPK are also known to phosphorylate T286, also leading to ubiquitin-dependent degradation of CCND1 [6, 7].

Deregulated expression of CCND1 in cancer can be caused by several mechanisms. In mantle cell lymphoma (MCL), the *CCND1* gene located on chromosome 11 is usually rearranged to a strong enhancer in the immunoglobulin heavy-chain (*IGH*) locus on chromosome 14, leading to increased *CCND1* transcription and CCND1 protein levels [8, 9]. Increased CCND1 levels also occur due to genomic deletions or point mutations in the 3′ UTR, which results in shorter, more stable transcripts [10, 11]. Experimental models that expressed a non-degradable CCND1 variant, in which T286 was substituted by alanine, or expression of an alternatively spliced CCND1b isoform, which lacks T286, have resulted in predominantly nuclear CCND1 expression and cellular transformation [12, 13]. In addition, aberrant activation of AKT and mTOR signaling results in down-regulation of GSK3B, also leading to reduced phosphorylation-dependent proteolysis and increased CCND1 protein levels [14].

Mantle cell lymphoma (MCL) is an incurable B-cell malignancy that frequently develops resistance to conventional chemotherapy and has a prognosis with a median overall survival of approximately 1–2 years after relapse [15, 16]. Recent treatment advances using the FDA-approved drug ibrutinib, which targets the B-cell antigen receptor (BCR) signaling molecule Bruton's tyrosine kinase (BTK), have produced durable responses in MCL [17]. However, one-third of MCL patients are ibrutinib-resistant, and even ibrutinib-sensitive patients eventually acquire resistance to the drug [17, 18]. The mechanisms underlying primary resistance to ibrutinib are not well understood. Recent studies have begun to provide some clues about potential mechanisms of primary ibrutinib resistance, including activation of the alternative NF-kB [19], ERK1/2 or AKT signaling pathways [20]. Mechanisms of acquired resistance to ibrutinib in patients who initially responded to the drug but then relapsed have also been reported, including recurrent mutations of the enzyme active site in BTK (C481S) or its substrate phospholipase C gamma 2 (PLCG2) [18, 21, 22]. These studies suggest that multiple mechanisms likely contribute to ibrutinib resistance in MCL.

Recent large-scale genomic studies of MCL have identified a hotspot for recurring somatic mutations in exon 1 of *CCND1* in 18–35% of the cases, likely arising through somatic hypermutation [23–25]. However, little is known about the functional role of these mutations in MCL. This study investigated the functional consequences of *CCND1* mutation on protein stability and sensitivity of MCL cells to ibrutinib therapy. The three most frequent *CCND1* mutations (E36K, Y44D and C47S) were cloned and expressed in MCL cell lines or HEK-293T cells. CCND1 protein stability and interaction with GSK3B were evaluated by cyclohexamide treatment and immunoprecipitation, respectively. Subcellular localization of the mutant CCND1 protein was determined by cell fractionation and immunofluorescence. In addition, primary MCL tumors with *CCND1* mutations were examined for CCND1 protein stability and sensitivity to ibrutinib. These studies have uncovered an important role for *CCND1* mutations in deregulating protein turnover, and a potential role in resistance to ibrutinib in some MCL tumors.

## RESULTS

### *CCND1* mutations increased CCND1 protein levels through defective proteolysis

**Figure 1:**
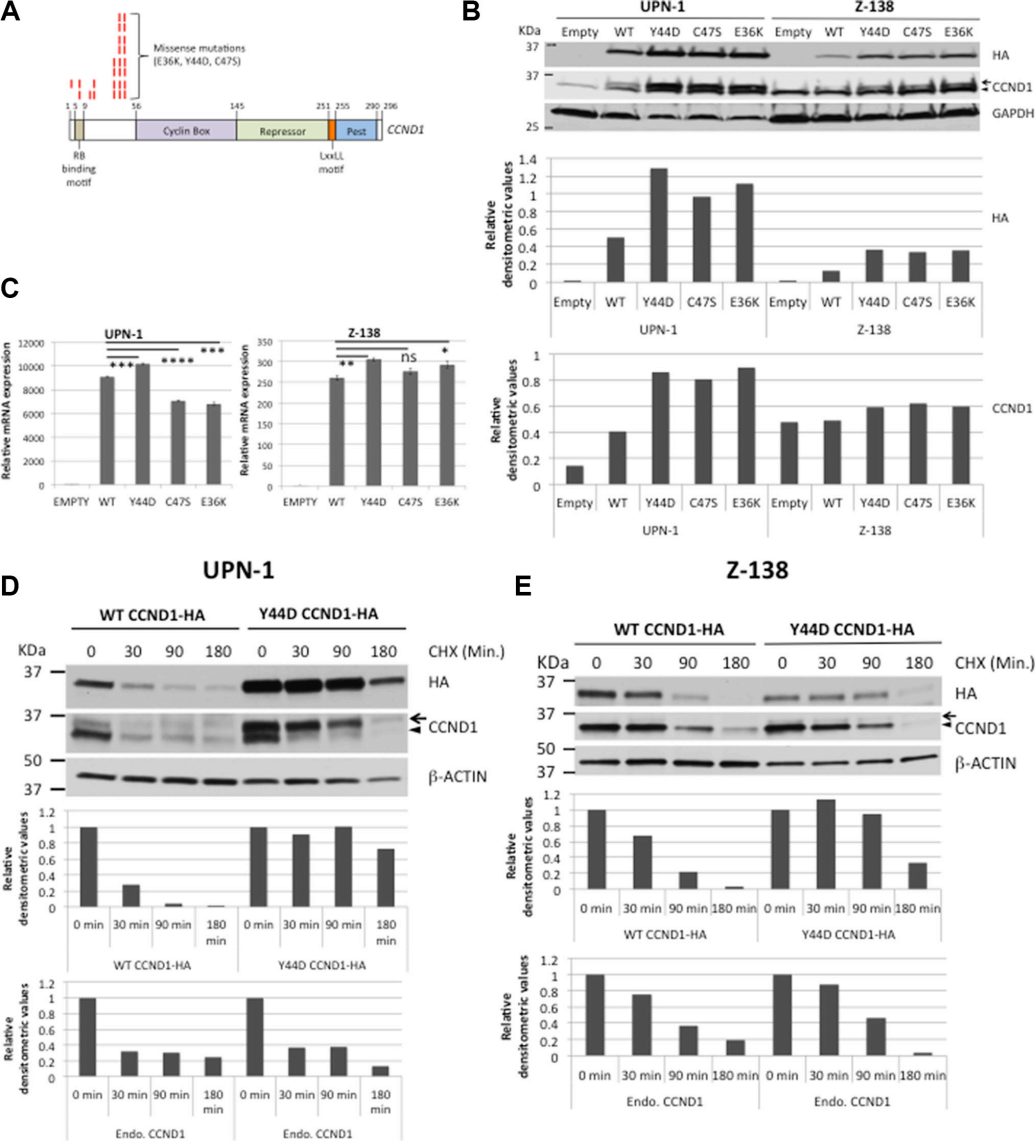
*CCND1* mutations increase protein levels (**A**) Diagram shows recurring point mutations (red bars) in the N-terminus of CCND1 protein. (**B**) Immunoblot analysis of WT and mutant CCND1 expression. UPN-1 or Z-138 cells were transduced with WT or mutant CCND1-HA and selected for stable expression by hygromycin. Cell lysates (10 μg per lane) were separated by SDS-PAGE gel and immunoblotted with indicated antibodies. Arrow indicates a mobility shift of the CCND1-HA protein. Arrowhead indicates endogenous CCND1. Bar graphs below the immunoblot show relative densitometric values of indicated bands after normalization to GAPDH loading controls. (**C**) Quantitative PCR analysis of mRNA expression of WT and mutant *CCND1*. Cell lines generated as described in (B) and mRNAs were harvested for quantitative PCR analysis of CCND1-HA mRNA expression. Shown are the means of mRNA expression levels after normalization to GAPDH signals from four independent amplification experiments. Error bars, SD. ****P < 0.0001; ***P < 0.001; **P < 0.01; *P < 0.05 by a two-tailed Student's *T-test*. (**D**, **E**) UPN-1 or Z138 cells expressing WT or Y44D CCND1-HA were treated with 10 μM of cyclohexamide (CHX) for indicated times and 10 μg of cell lysates per lane were prepared for immunoblot analysis with indicated antibodies. Arrow, CCND1-HA protein. Arrowhead, endogenous CCND1. Bar graphs below immunoblots in (D, E) show relative densitometric values of indicated bands from the blots after normalization to time zero control samples.

To study the role of *CCND1* somatic mutations, the *CCND1* exon 1 of eight MCL cell lines was sequenced and found to have the germ-line sequence (data not shown). Therefore, site-directed mutagenesis was used to generate the three most frequent mutations, E36K, Y44D and C47S, as previously reported (Figure 1A) [19, 23–25]. Hemagglutinin (HA)-tagged wild type (WT) or mutant *CCND1* cDNA was cloned into a retroviral vector and expressed in the MCL cell lines UPN-1, Z-138 and JEKO-1. An empty vector was used as a negative control. After establishing stably transduced cells by hygromycin selection, equal numbers of cells from each culture were harvested and mRNA and total protein lysates were prepared. Anti-HA antibody was used to assess exogenous CCND1 protein expression by immunoblot analysis. All three mutants showed increased protein expression compared to the WT counterparts in all three MCL cell lines (Figure 1B, Supplementary Figure S1A). In Supplementary Figure S1A, JEKO-1 cells that expressed the non-degradable T286A *CCND1* mutant [5] were also included for comparison. Compared to WT, mutant CCND1 proteins did not affect the kinase function of CDK4, as determined by phosphorylation of the CDK4 substrate Rb in JEKO-1 cells (Supplementary Figure S1B). To determine whether increased protein expression was due to increased transcription, mRNA expressed from WT and mutant *CCND1* was compared by real-time quantitative PCR (qPCR) using primers specific for the exogenous *CCND1-HA* mRNA (Figure 1C). There was no significant increase in mutant mRNA levels compared to that of WT samples in both UPN-1 and Z-138 cells (Figure 1C). Next, we determined whether increased protein levels were due to deregulated protein turnover. We treated WT or mutant *CCND1*-expressing cells with cyclohexamide (CHX) to inhibit *de novo* protein synthesis, and CCND1 proteolysis was examined by immunoblot analysis. In both UPN-1 and Z-138 cells, while more than 50% of WT CCND1 was degraded as early as 90 min after CHX, little reduction in Y44D CCND1 levels was observed during the same period (Figure 1D, 1E). Similar stability was also observed the C47S mutant in UPN-1 cells under the same experimental conditions (Supplementary Figure S2). Taken together, we concluded that *CCND1* mutations increased protein, but not mRNA, levels, and this was likely due to defective proteolysis.

### *CCND1* mutations interfere with T286 phosphorylation

**Figure 2:**
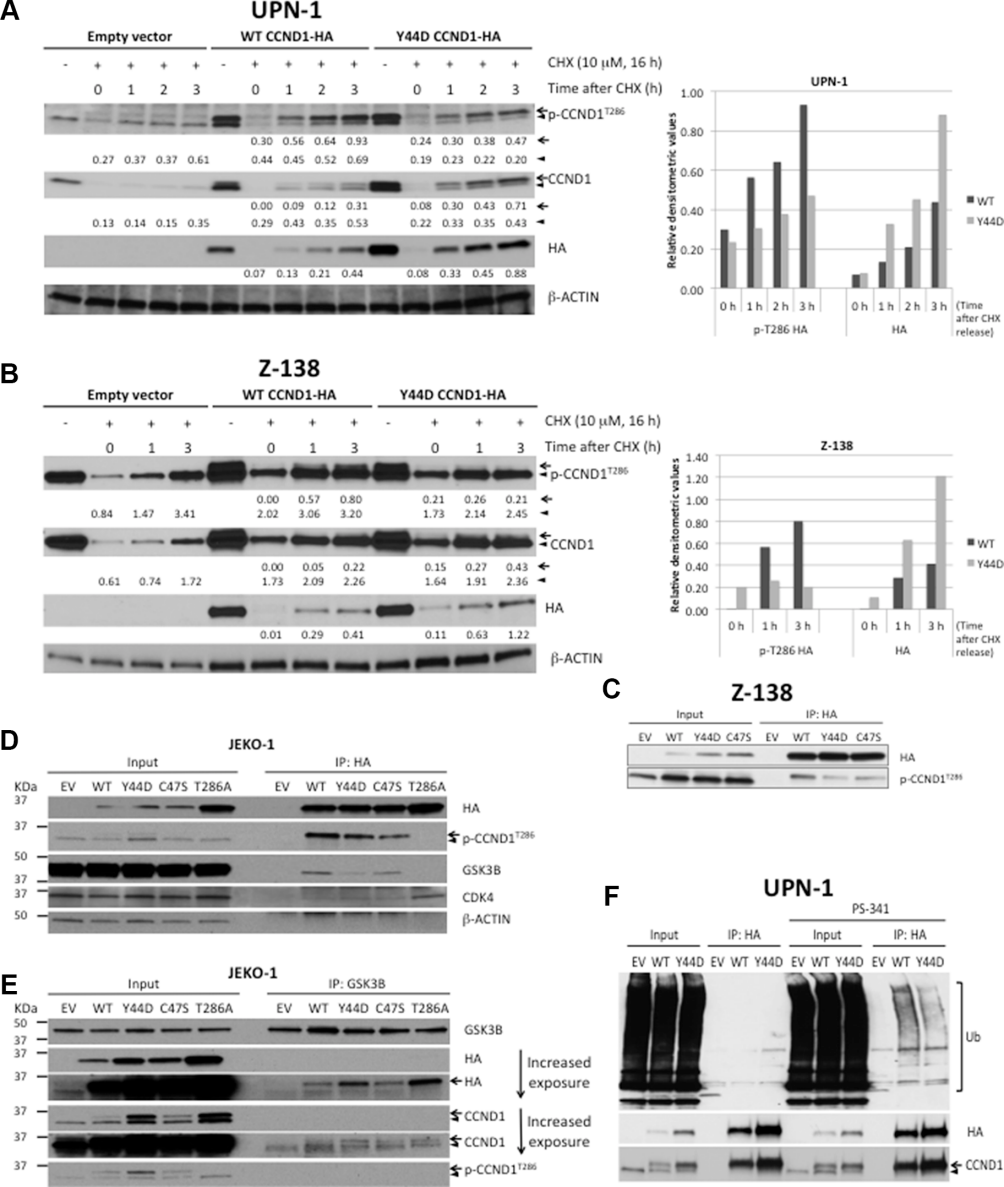
*CCND1* mutations interfere with T286 phosphorylation and protein ubiquitination (**A**, **B**) UPN-1 or Z138 cells expressing WT, Y44D CCND1-HA, or an empty vector control were treated with 10 μM of cyclohexamide (CHX) for 16 hours and released to normal growth medium for indicated times. Cell lysates (10 μg per lane) were prepared for immunoblot analysis with indicated antibodies. Arrow, CCND1-HA protein. Arrowhead, endogenous CCND1. Numbers below the blots are relative densitometric values of corresponding bands after normalization to β-ACTIN loading controls. Graphical representation of selected densitometric values was shown in the bar graphs on the right. (**C**) Z-138 cells expressing an empty vector (EV), WT, or mutant CCND1-HA were immunoprecipitated with HA antibody and immunoblotted with indicated antibodies. Lysates before immunoprecipitation were used as input samples. (**D**, **E**) Analysis of mutant CCND1 interaction with GSK3B. (D) JEKO-1 cells expressing an empty vector (EV), WT, or mutant CCND1-HA were immunoprecipitated with HA antibody and immunoblotted with indicated antibodies. (E) JEKO-1 cell lines described in (D) were immunoprecipitated with GSK3B antibody and immunoblotted with indicated antibodies. Arrow, CCND1-HA protein. Arrowhead, endogenous CCND1. (**F**) Y44D *CCND1* mutation affects ubiquitin (Ub)-dependent degradation. UPN-1 cells that stably expressed an empty vector (EV) control, WT or Y44D CCND1-HA were treated with 5 nM of the proteasome inhibitor PS-341 for 2 h. Treated cells were lysed in 2% SDS and heated at 95°C for 5 min. Lysates were subsequently immunoprecipitated with HA antibodies and Ub conjugates on CCND1 were examined by immunoblot analysis with anti-Ub antibody.

Since phosphorylation of T286 is required for proteasome-mediated CCND1 turnover [5], we next examined the effect of CCND1 mutations on this phosphorylation. Immunoblot analysis of T286 phosphorylation levels taken directly from on-going cell cultures was, however, confounded by the higher steady-state levels of mutant CCND1 proteins (Supplementary Figure S1C). As an alternative approach, we evaluated T286 phosphorylation in cells that were treated with CHX over night to allow complete degradation of CCND1, and then returned back to normal growth conditions for renewed protein synthesis. Under this experimental condition, T286 phosphorylation of newly synthesized Y44D CCND1 was markedly reduced in both UPN-1 and Z-138 cells as compared to phosphorylation of WT CCND1 (Figure 2A, 2B). Reduced T286 phosphorylation of Y44D CCND1 was not due to decreased protein levels, as the mutant protein levels accumulated faster than WT protein over the same time period after CHX treatment and release (Figure 2A, 2B). Because of the low expression levels of CCND1-HA in Z-138 cells (Figure 1B and Figure 2B), to enhance detection of T286 phosphorylation, CCND1-HA was immunoprecipitated with the HA-specific antibody followed by immunoblotting with the phospho-T286 CCND1 antibody. T286 phosphorylation levels were markedly reduced in both Y44D and C47S CCND1 immunoprecipitates, as compared to phospho-T286 signals on WT CCND1 (Figure 2C). These data indicate that these N-terminal *CCND1* mutations interfere with T286 phosphorylation at the C-terminus of CCND1.

Reduced T286 phosphorylation on mutant CCND1 prompted us to evaluate the interaction of the T286 phosphorylating enzyme GSK3B with WT and mutant CCND1 using the co-immunoprecipitation (co-IP) method. Using anti-HA antibody to immunoprecipitate CCND1-HA proteins in JEKO-1 cells, we detected little difference between WT and mutant proteins on binding with known interacting partners such as CDK4 (Figure 2D). In contrast, GSK3B was found to co-precipitate more with WT than with Y44D or C47S mutants (Figure 2D). Surprisingly, GSK3B was not detectable in T286A CCND1-HA pull-down despite the fact that this mutant had WT sequence at the N-terminus (Figure 2D). To confirm differential binding of WT and mutant CCND1 to GSK3B, we performed a reverse co-IP experiment using an anti-GSK3B antibody. Figure 2E shows that all three mutants co-migrated with GSK3B without reduced binding, which is inconsistent with the results observed in the HA pull-down experiments. Despite the apparent discrepancy between the forward and reverse IP experiments, which could reflect the accessibility of the C-terminally tagged HA epitope (see Discussion), our data appear to indicate little effect of Y44D and C47S mutations on CCND1-GSK3B interaction.

Because T286 phosphorylation increases the ubiquitination of CCND1 protein [5], we next determined whether *CCND1* mutations affected protein ubiquitination. UPN-1 cells that stably express empty vector, WT or Y44D CCND1-HA were briefly treated with 5 nM of the proteasome inhibitor PS-341 (bortezomib) for two hours to enhance detection of ubiquitination. Treated cells were lysed in 2% SDS and heated at 95°C for five minutes to disrupt potential interactions of ubiquitin (Ub)-conjugated proteins that might co-precipitate with CCND1-HA. These lysates were subsequently immunoprecipitated with HA antibodies and Ub conjugates on CCND1 were examined by immunoblot analysis with anti-Ub antibody. As expected, WT CCND1 (both endogenous and exogenous protein) levels in the input accumulated after two hours of bortezomib treatment (Figure 2F). In contrast, little accumulation of mutant CCND1-HA was detected under the same treatment conditions (Figure 2F), consistent with deregulated proteolysis of mutant CCND1 as shown above. In the immunoprecipitates, more Y44D than WT CCND1 protein levels were detected, consistent with their respective CCND1-HA levels in the inputs (Figure 2F). However, Ub conjugate signals on Y44D CCND1 were less than that on WT CCND1 (Figure 2F). These results indicate that the Y44D mutation increases CCND1 protein stability through disruption of the Ub-proteasome pathway.

### Y44D and C47S mutants preferentially localize to the nucleus

**Figure 3:**
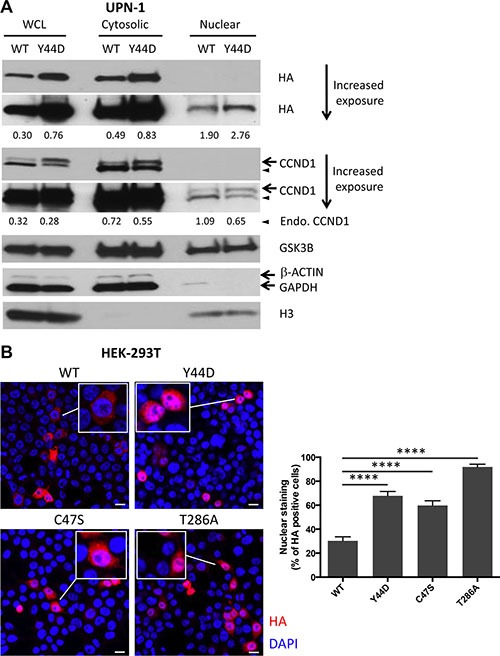
Subcellular localization of CCND1 mutants (**A**) Cytosolic and nuclear extracts were prepared as described in Materials and Methods from UPN-1 cells that stably expressed WT or Y44D CCND1. The extracts (30 μg per lane) were immunoblotted with indicated antibodies. β-ACTIN or GAPDH and histone H3 were used to confirm cytosolic and nuclear fractions, respectively. Numbers below the blots are relative densitometric values of corresponding bands after normalization to β-ACTIN (for WCL and Cytosolic) or histone H3 (for Nuclear) loading controls. WCL, whole cell lysates; Endo., endogenous. (**B**) Subcellular distribution of WT and mutant CCND1-HA proteins, which were transiently expressed in HEK-293T cells, was evaluated by immunofluorescence. Shown are representative confocal immunofluorescence images of WT and mutant CCND1-expressing cells stained with anti-HA antibody (red) followed by nuclear staining with DAPI (blue). Scale bars, 20 μm. Bar graphs show the percentages of HA positive cells that have HA staining in the nucleus from two separate experiments. Error bars, SEM; ****P < 0.0001 by a two-tailed Student's *T-test*. Approximately 150 HA positive cells from each group were counted.

The T286A mutant protein was found predominantly in the nucleus because this mutation disrupted nuclear export of CCND1 [4]. To determine the cellular localization of CCND1 with N-terminal mutations, which also interfere with T286 phosphorylation, we performed cell fractionation experiments and compared WT and Y44D CCND1-HA protein levels in the nuclear extracts of UPN-1 cells. Cytosolic and nuclear fractions were confirmed by immunoblotting with the cytosolic markers β-ACTIN or GAPDH and nuclear marker histone H3. While WT and Y44D CCND1-HA proteins were detected in both the cytosolic and nuclear fractions, Y44D CCND1 protein levels were consistently higher than that of WT CCND1 in both fractions (Figure 3A). As an alternative approach, we used immunofluorescence to evaluate the subcellular distribution of WT and mutant CCND1-HA proteins expressed in HEK-293T cells, which were chosen for their large cytoplasmic surface area to facilitate data analysis. Using fluorescently labeled anti-HA antibody, we demonstrated that the Y44D and C47S mutant proteins preferentially localized to the nucleus as compared to WT CCND1 protein (Figure 3B). The T286A mutant was found predominantly in the nucleus, as expected from previous studies [4]. Together, we conclude that the Y44D and C47S *CCND1* mutation interferes with T286 phosphorylation and deregulates CCND1 nuclear export.

### *CCND1* mutations promote resistance to ibrutinib

**Figure 4:**
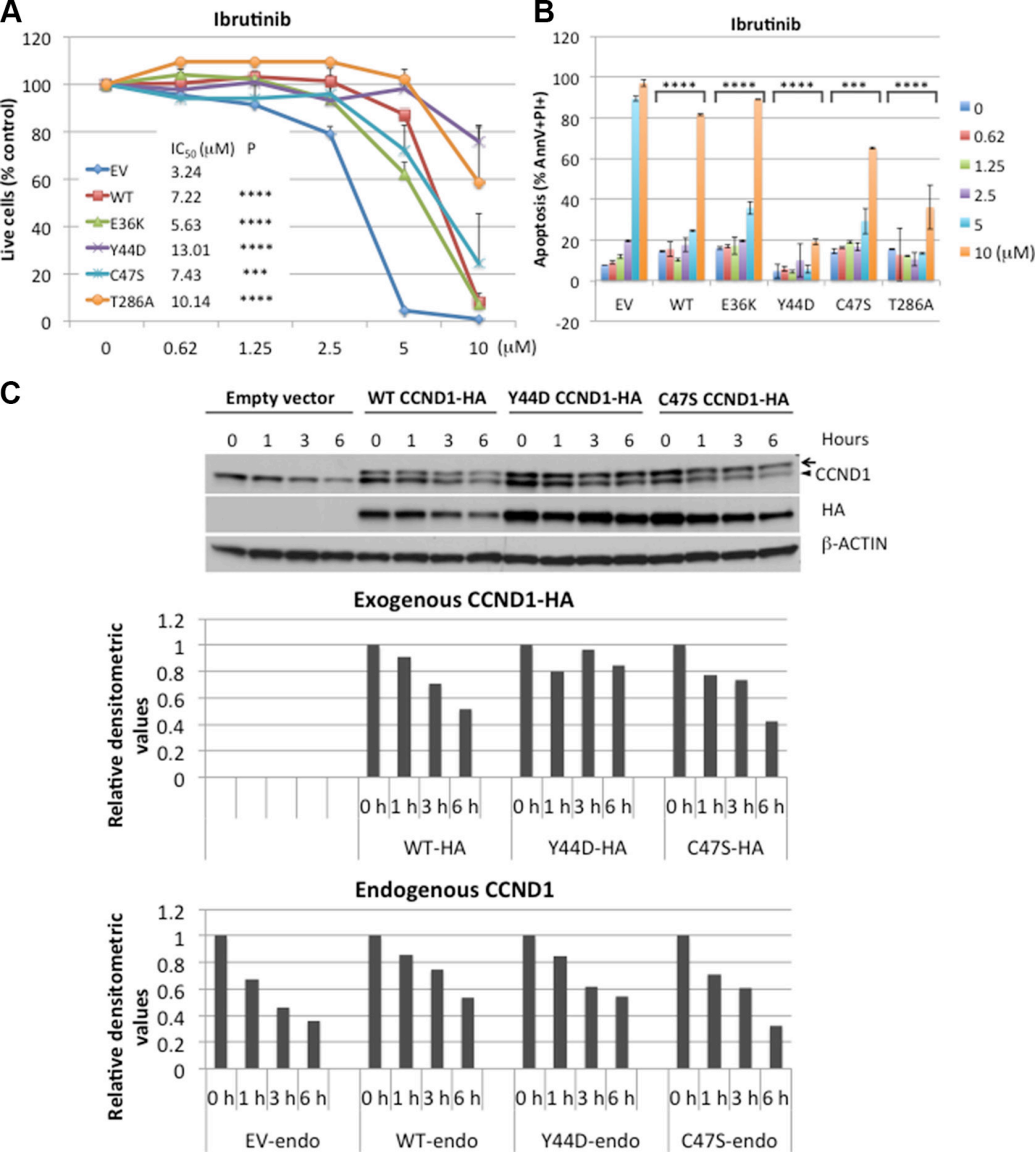
*CCND1* mutations promote resistance to ibrutinib (**A**) JEKO-1 cells expressing an empty vector (EV), WT, or mutant CCND1-HA were treated with indicated doses of ibrutinib for four days and propidium iodide (PI)-negative (viable) cells were assessed by flow cytometry. Shown are PI negative fractions normalized to control untreated samples. Fifty percent inhibition concentration (IC_50_) values were calculated by GraphPad Prism 7 software (GraphPad). (**B**) Apoptosis of JEKO-1 cells in the same experiment as described in A were assessed by Annexin V/PI staining on day 4. Shown are percentages of Annexin V+/PI+ cells. Line graphs or bar graphs show means of three independent experiments. Error bars, SD. Error bars where not visible fall within the points. ****P < 0.0001, ***P < 0.001 (two-way ANOVA). *P* values indicate significance levels for the effect of WT or mutants on ibrutinib sensitivity compared to empty vector (EV) control. (**C**) JEKO-1 cells expressing an empty vector (EV), WT, or mutant CCND1-HA were treated with 10 μM of ibrutinib for indicated times and 10 μg of cell lysates per lane were prepared for immunoblotting with indicated antibodies. Arrow, CCND1-HA protein. Arrowhead, endogenous CCND1. Bar graphs below the blot show relative densitometric values of indicated bands after normalization to time zero control samples.

Previous studies showed that CCND1 was essential for MCL survival [26, 27], and that ibrutinib treatment decreased CCND1 protein levels in sensitive, but not in resistant, MCL cell lines [20]. These findings prompted us to determine a potential correlation between CCND1 levels and ibrutinib sensitivity. Ibrutinib-sensitive JEKO-1 cells [19, 20], which stably expressed WT or mutant CCND1 (Supplementary Figure S1A), were treated with a single dose of increasing concentrations of ibrutinib for four days and analyzed for cell growth and apoptosis using flow cytometry. Ibrutinib significantly inhibited cell growth and induced apoptosis in a dose-dependent manner in control cells transduced with an empty vector. In contrast, WT and mutant CCND1-expressing cells were more resistant up to 5 μM of ibrutinib. At 10 μM, ibrutinib was toxic to WT, E36K and C47S mutants, but less so to Y44D and T286A mutants (Figure 4A, 4B). To confirm that the rescue effect of exogenously expressed CCND1 protein was specific to loss of BTK activity, we used a short-hairpin RNA (shRNA) to deplete BTK in JEKO-1 cells that expressed CCND1 variants (Supplementary Figure S3A). As expected, BTK depletion was toxic in JEKO-1 cells that expressed an empty vector (Supplementary Figure S3B). However, BTK shRNA-mediated toxicity in JEKO-1 cells was rescued by exogenous WT or mutant CCND1 (Supplementary Figure S3B), consistent with results shown in Figure 4A, 4B.

We next examined the effect of ibrutinib on expression of CCND1 protein levels. JEKO-1 cells expressing WT or mutant CCND1 were treated with 10 μM of ibrutinib for up to six hours and CCND1 levels were determined by immunoblot analysis. In a time-dependent manner, ibrutinib decreased the protein levels of WT and C47S mutant but not of Y44D mutant (Figure 4C), which appeared to correlate with the resistance of the Y44D mutant at high doses of the drug. These data indicate that increased CCND1 levels promote ibrutinib resistance and certain *CCND1* mutations can provide sustained protection from high doses of ibrutinib.

**Figure 5:**
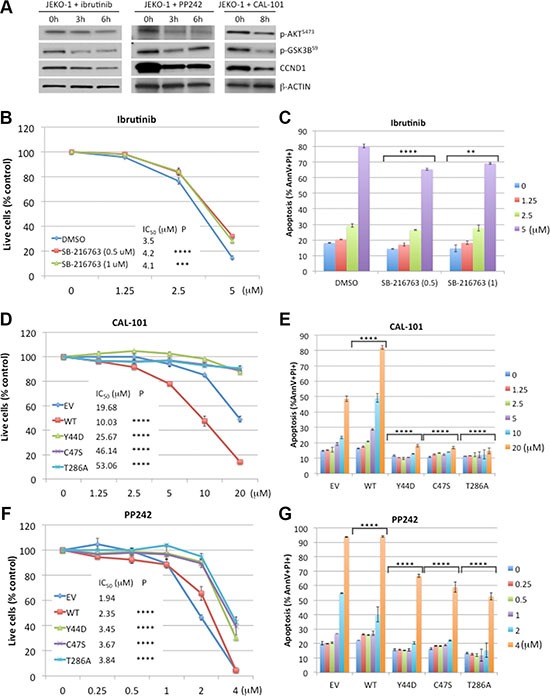
*CCND1* mutations promote resistance to CAL-101 and PP242 (**A**) Immunoblot analysis of JEKO-1 cells treated with ibrutinib (10 μM), PP242 (4 μM) or CAL-101 (10 μM) for indicated times and immunoblotted (30 μg of lysate per lane) with indicated antibodies. (**B**, **C**). SB-216763 rescues ibrutinib toxicity. JEKO-1 cells were co-treated with indicated doses of ibrutinib and 0.5 μM or 1 μM of SB-216763. Viable, PI-negative cells (B) or apoptotic cells (C) were assessed by flow cytometry on day 4. (**D**–**G**) JEKO-1 cells expressing an empty vector (EV), WT, or mutant CCND1-HA were treated with indicated doses of CAL-101 (D, E) or PP242 (F, G) and live cells (D, F) or apoptotic cells (E, G) were analyzed by flow cytometry on day 4. Shown in line graphs are PI negative fractions normalized to control untreated samples. Line graphs or bar graphs show means of three independent experiments. Error bars, SD. Error bars where not visible fall within the points. ****P < 0.0001, ***P < 0.001, **P < 0.01 (two-way ANOVA). *P* values indicate significance levels for the effect of SB-216763 (B, C), compared to DMSO, or of WT and mutants (D–G), compared to empty vector (EV) control, on sensitivity to increasing concentrations of the indicated drugs.

Defective T286 phosphorylation-dependent degradation of the Y44D CCND1 mutant and its protection of JEKO-1 cells against ibrutinib suggests a role for GSK3B in ibrutinib toxicity. To test this hypothesis, we used immunoblot analysis to evaluate GSK3B activity in JEKO-1 cells treated with ibrutinib. In a time-dependent manner, ibrutinib reduced the inhibitory serine-9 phosphorylation of GSK3B [28, 29], resulting in increased activity of this kinase (Figure 5A). Consistent with this finding, the activity of AKT, which phosphorylates GSK3B on serine-9 [30], was also reduced (Figure 5A). These results indicate that ibrutinib antagonizes the regulatory role of AKT, leading to re-activation of GSK3B which, in turn, mediates CCND1 degradation. To confirm that ibrutinib toxicity was GSK3B dependent, JEKO-1 cells were treated with increasing doses of ibrutinib in the presence of the GSK3B inhibitor SB-216763 [31]. Compared to DMSO-treated controls, addition of SB-216763 at both 0.5 and 1 μM partially rescued the cells from ibrutinib-mediated growth inhibition and apoptosis (Figure 5B, 5C). Treatment with the inorganic salt LiCl, which inhibits GSK3B [32], also rescued JEKO-1 cells from ibrutinib toxicity (Supplementary Figure S4). Thus, GSK3B kinase activity contributes to the cytotoxic effect of ibrutinib in JEKO-1 cells.

To further confirm the role of CCND1 mutations in interference with GSK3B-mediated T286 phosphorylation, we next investigated whether these mutations rescue cells from pharmacological agents that target the AKT-GSK3B pathway such as the PI3K inhibitor CAL-101 (idelalisib) [33] and the mTOR kinase inhibitor PP242 [34]. As expected, these drugs reduced AKT activity, re-activated GSK3B by decreasing serine-9 phosphorylation, and down-regulated CCND1 levels (Figure 5A). Similar to ibrutinib, both CAL-101 and PP242 demonstrated dose-dependent toxicity in control or WT CCND1-HA expressing JEKO-1 cells. However, overexpression of Y44D, C47S or T286A CCND1 was sufficient to rescue the cells from growth inhibition and apoptosis induced by increasing concentrations of CAL-101 or PP242 (Figure 5D–5G). Altogether, these data indicate that *CCND1* mutations promote resistance to GSK3B activating agents such as ibrutinib, CAL-101 or PP242.

### Analysis of *CCND1* mutations in primary MCL tumors

**Figure 6:**
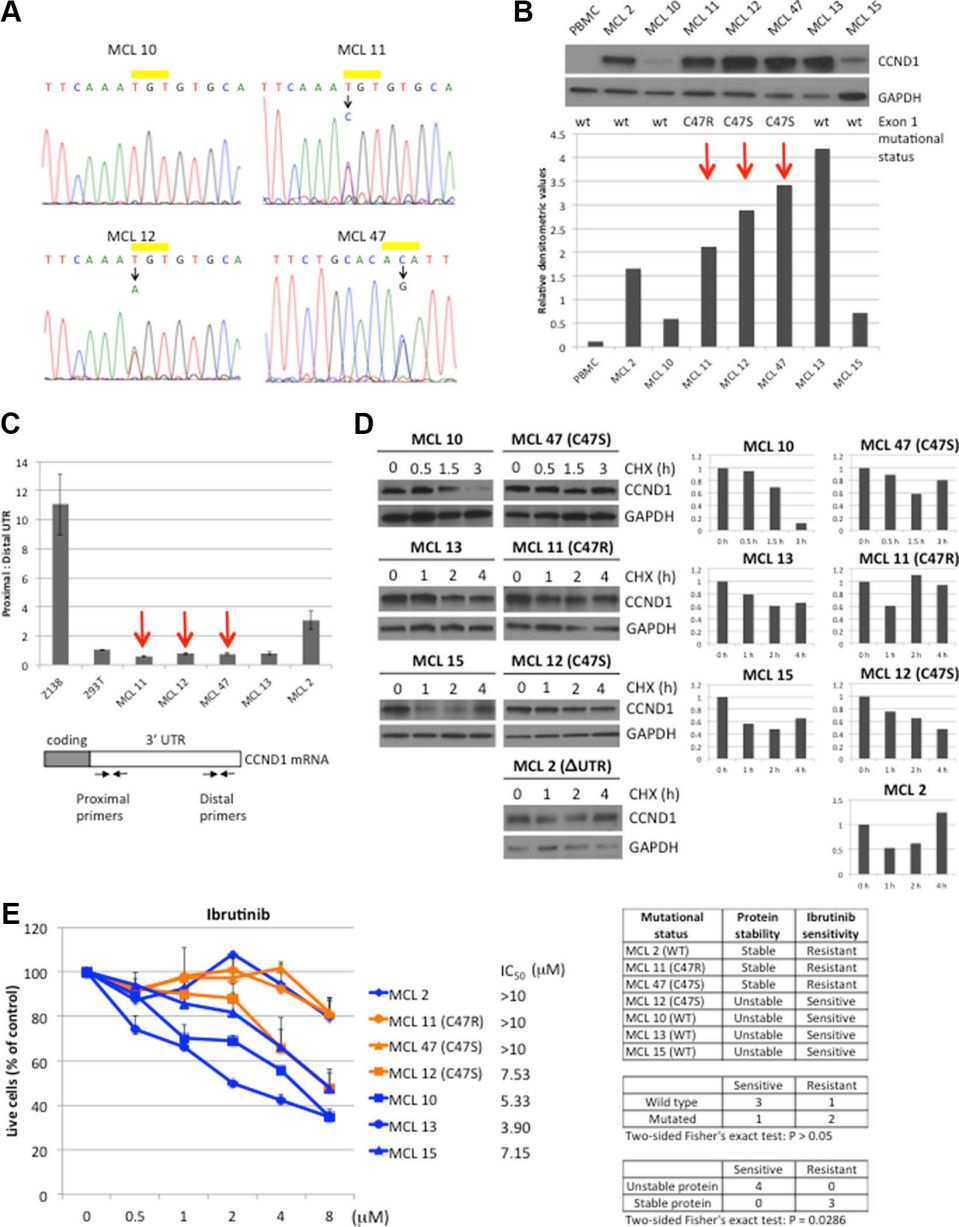
*CCND1* mutations in primary MCL (**A**) Sequencing chromatograms of CCND1 point mutations in MCL samples. WT and mutated CCND1 alleles on residue cysteine-47 (yellow bar) from MCL tumors 10, 11, 12 and 47 are shown. For MCL 47, complementary sequence from the reverse primer is shown. (**B**) CCND1 expression in MCL tumors. Lysates (10 μg per lane) from indicated MCL tumors were immunoblotted with indicated antibodies. Peripheral blood mononuclear cells (PBMC) were used as negative control for CCND1 expression. Bar graphs show relative densitometric values of CCND1 expression from the immunoblot after normalization to GAPDH loading control. Red arrows highlight the *CCND1* mutated samples. (**C**) Analysis of *CCND1* truncated 3′UTR transcript in MCL tumors. The relative abundance between the full-length and truncated 3′UTR was determined by using proximal and distal primers (see primer locations in the diagram of the *CCND1* mRNA transcript). (**D**) CCND1 stability in MCL tumors. Indicated MCL tumors were treated with 10 μM of cyclohexamide (CHX) for indicated times and 10 μg of cell lysates were prepared for immunoblot analysis with indicated antibodies. Bar graphs show relative densitometric values of CCND1 expression from corresponding blots after normalization to GAPDH. (**E**) Sensitivity of MCL tumors to ibrutinib. Indicated MCL tumors were treated with indicated doses of ibrutinib for three days and metabolically active cells were assessed by CellTiter-Glo Luminescent assay (Promega). Shown are fractions of luminescence signals from metabolically active cells normalized to untreated samples. Line graphs show mean values from three independent experiments. Error bars, S.E.M. IC_50_ values were calculated by GraphPad Prism 7 software. The table summarizes the ibrutinib response with respect to *CCND1* mutation status or protein stability. Correlation of either mutation status or protein stability with ibrutinib sensitivity was analyzed by the two-sided Fisher's exact test.

To investigate the role of *CCND1* mutations in primary MCL tumors, the sequence of *CCND1* exon 1 was determined by Sanger sequencing in 16 primary tumors. The tumor content in these samples was confirmed to have greater than 90% CD19+, CD5+ lymphoma cells by flow cytometry (data not shown). Three samples from patients who did not received ibrutinib therapy at the time of sample collection were found to carry single C47S or C47R heterozygous mutations (Figure 6A). Immunoblot analysis of seven samples with sufficient tumor materials showed that MCL tumors with *CCND1* mutations expressed more CCND1 protein than WT samples, except for sample MCL 13 (Figure 6B). To examine whether increased CCND1 protein levels in these mutant cases was due to the presence of more stable, 3′ UTR-truncated transcripts, we used a previously-described RT-PCR assay [10] to compare full-length and 3′ UTR-truncated mRNA levels in these samples. While Z-138 cells were confirmed to predominantly express 3′ UTR-truncated *CCND1* mRNA as previously reported [10], all three *CCND1* mutated samples expressed the full-length mRNA, as did control HEK-293T cells (Figure 6C). One sample (MCL 2) with WT *CCND1* exon 1 was found to express UTR-truncated transcripts (Figure 6C). Therefore, we conclude that increased CCND1 protein accumulation in the mutant cases was not due to expression of stable UTR-truncated *CCND1* mRNAs.

To examine whether the increased protein level in MCL tumors with *CCND1* mutations was due to deregulated protein turnover, MCL cells were treated with CHX and the rate of CCND1 proteolysis was evaluated by immunoblot analysis. In contrast to WT CCND1 samples examined (MCL 10, 13 and 15), which showed CCND1 degradation after three hours in CHX, two of three *CCND1* mutant samples (MCL 11 and 47) had a prolonged half-life (Figure 6D). Interestingly, sample MCL 2, which expressed WT CCND1, also showed less CCND1 degradation (Figure 6D), likely due to unknown mutations that deregulate CCND1 proteolysis. Thus, *CCND1* mutations may affect protein stability in some primary MCL tumors and correlate with increased protein levels in these samples.

We next compared the sensitivity of MCL tumors that express WT or mutant *CCND1* to ibrutinib. In a dose-dependent manner, ibrutinib reduced cell viability in all three WT *CCND1* MCL samples (MCL 10, 13, and 15) after 72 hours (Figure 6E). In contrast, two of the three mutated samples (MCL 11 and 47) were more resistant to ibrutinib. Interestingly, MCL 2 was also resistant to the drug, which appeared to correlate with increased CCND1 protein stability observed in this sample. The difference in ibrutinib response between WT and mutated samples was not statistically significant, likely due to the small sample size. However, there was a significant difference in response to ibrutinib between samples with different CCND1 protein stability. These results indicate that *CCND1* mutations or other mechanisms that increase CCND1 protein stability and/or levels contribute to the resistance of MCL tumors to ibrutinib.

## DISCUSSION

High-throughput sequencing of the MCL genome has consistently revealed *CCND1* as the second most frequently mutated gene after the ataxia telangiectasia mutated (ATM) gene [23–25]. We found that these recurrent mutations, which are located in the N-terminus of CCND1, interfere with T286 phosphorylation, leading to deregulated CCND1 turnover and increased protein levels. In addition, these mutations increased the resistance of MCL to ibrutinib, highlighting their relevance to this emerging therapy.

Many lines of evidence suggest that CCND1 translocation alone is not sufficient and additional genetic lesions are required for MCL development. For example, the Emu-CCND1 transgenic mouse, which mimics the t(11;14) translocation, developed lymphoma only after crossing with transgenic mice that expressed an established oncogene such as *MYC* [35, 36]. Alternatively, B-cell lymphoma also developed when crossing the Emu-CCND1 mouse with transgenic mice that had other genetic aberrations observed in MCL, such as BIM-deficient [37] or ATM-deficient mice [38]. Interestingly, the Emu-T286A CCND1 transgenic mice, which constitutively expressed nuclear mutant CCND1, developed lymphoma without introducing a second genetic hit [13]. Although the B-cell lymphomas that developed in these mice had a mature B-cell phenotype like human MCL, the T286A mutation has been observed only rarely in MCL and whether this mouse model recapitulates human MCL remains uncertain. Our data demonstrate that N-terminal *CCND1* mutations interfere with T286 phosphorylation and promote CCND1 nuclear localization, which are reminiscent of the T286A CCND1 mutant phenotype. The frequent occurrence of these N-terminal *CCND1* mutations indicates that defective T286 phosphorylation may be more common in MCL than previously thought. Our findings thus support the oncogenic role of defective T286 phosphorylation, as postulated by Diehl and colleagues [39].

How do N-terminal CCND1 mutations affect the C-terminal T286 phosphorylation? Our initial hypothesis was that these mutations may affect docking of GSK3B and, thus, subsequent phosphorylation. Although co-IP experiments using an anti-HA antibody showed less binding of GSK3B to Y44D and C47S mutants, reverse co-IP experiments using anti-GSK3B antibody did not confirm this result. In contrast, GSK3B pull-down showed little effect of Y44D or C47S mutants on interaction with GSK3B. An important clue from this apparent discrepancy between the forward and reverse IP experiments was the absence of GSK3B signals in the T286A-HA pull-down (Figure 2D). However, T286A-HA signals were readily detectable in the immunoprecipitates of GSK3B pull-down, which confirmed GSK3B-T286-HA interaction (Figure 2E). These observations indicate that, unlike in the WT CCND1-HA pull-down, the anti-HA antibody did not recognize the C-terminally tagged HA epitope of the T286A mutant in the complex with GSK3B. Therefore, we speculate that reduced GSK3B signals in the immunoprecipitates of Y44D or C47S pull-down, as compared to WT, may be due to reduced binding of the anti-HA antibody to the C-terminally tagged HA epitope in these mutants. Re-cloning of CCND1 constructs with N-terminally tagged HA is, therefore, necessary to test this possibility and to clarify whether CCND1 mutations affect GSK3B docking.

Despite the efficacy of ibrutinib in MCL, one-third of the patients do not respond to this therapy and acquired resistance to this drug is almost universal [17, 40]. A better understanding of why some patients respond well, but others do not, is needed. Sustained PI3K/AKT signaling or increased activation of the alternative NF-kB pathway have been proposed as potential mechanisms that may compensate for the BCR—BTK—NF-kB axis, which is targeted by ibrutinib [19, 20]. Similar to agents that target AKT signaling (such as CAL-101 and PP242), ibrutinib can also inhibit AKT activity, leading to activation of GSK3B and increased phosphorylation-dependent proteolysis of CCND1. The N-terminal CCND1 mutations that increase protein stability by evading GSK3B-mediated phosphorylation provide another distinct mechanism of resistance to such therapies.

Two out of three MCL tumors with mutated *CCND1* were resistant to ibrutinib. However, sample MCL 12, which also carried the C47S mutation, was sensitive to this drug. The mutant CCND1 protein in this sample appeared less stable than CCND1 with the same mutation from sample MCL 47, as revealed by the CHX experiments (Figure 6D). In addition, although *in vitro* overexpression of C47S CCND1 was sufficient to confer ibrutinib resistance in JEKO-1 cells, protection from ibrutinib toxicity was less robust than the Y44D mutation. These observations suggest that *CCND1* mutations may provide different levels of protection from ibrutinib toxicity, depending on the cellular context and tumor heterogeneity. Conversely, sample MCL 2 had WT *CCND1* but was resistant to ibrutinib. Interestingly, this sample displayed more stable CCND1 protein, suggesting other mechanisms that may contribute to CCND1 stability in addition to the N-terminus point mutations. Because of the small sample size in the present study, further validation of our findings in more MCL tumors will be required to confirm the correlation between *CCND1* mutations and ibrutinib resistance.

In summary, these findings uncovered residues previously not known to play a role in the regulation of CCND1 turnover. Our data demonstrate that single N-terminal CCND1 mutations stabilize and increase protein levels, which contributes to ibrutinib resistance. This study suggests that *CCND1* mutations may be a useful biomarker for ibrutinib insensitivity. Further studies using a larger sample size are needed to evaluate this potential clinical utility.

## MATERIALS AND METHODS

### Cell lines and culture conditions

Human MCL lines UPN-1, Z-138 and JEKO-1 were kindly provided by Dr. Louis Staudt. UPN-1 cells were confirmed to carry the cyclinD1/IGH fusion gene and other chromosomal rearrangements, as previously described [41], by metaphase fluorescence *in situ* hybridization (FISH) analysis (data not shown). Z-138 cells were confirmed to express truncated CCND1 3′UTR transcripts as described [10] (Figure 6C). JEKO-1 cells were not authenticated. Cells were cultured in RPMI-1640 medium (Life Technologies, Grand Island, NY) supplemented with 10% fetal bovine serum and penicillin/streptomycin and maintained in a humidified, 5% CO2 incubator at 37°C.

### Primary MCL preparation

Viably cryo-preserved MCL cells were obtained from the tumor bank of the Pathology Department of City of Hope as de-identified samples after approval by the Institutional Review Board. Frozen cells were briefly thawed in 37°C water bath, washed in RPMI-1640 medium and cultured in RPMI-1640 medium supplemented with 20% fetal bovine serum and 200 Kunits/ml of DNAse I (Sigma, St. Louis, MO) for 15 minutes in 37°C CO2 incubator followed by washing. Cells were recovered overnight in CO2 incubator before experiments.

### Antibodies and chemicals

The following antibodies were used: HA (C29F4), Histone H3, pCCND1-T286 (D29B3), GSK3B (D5C5Z), pS9-GSK3B and p-AKT S473 (Cell Signaling Technology, Danvers, MA), CDK4, CCND1(M20), ACTIN, GAPDH, (Santa Cruz, Dallas, TX), The following chemicals were used: cyclohexamide, Lithium chloride (LiCl) (Sigma, St. Louis, MO) SB-216763, CAL-101, PP242, PS-341 (Selleck Chemicals, Houston, TX). Ibrutinib was obtained from commercial sources (Selleck Chemicals or MedKoo Biosciences, Inc. Chapel Hill, NC) or from Craig Thomas (NCI, Bethesda, MD) as a gift. Ibrutinib from these sources was confirmed to have similar killing activity on JEKO-1 cells (data not shown).

### Expression vectors

The retroviral expression vector pBMN-CCND1-HA-IRES-Hygro, encoding carboxy-terminus HA-tagged CCND1, was constructed by ligation of PCR-generated CCND1-HA products from the plasmid pRc/CMV-Cyclin D1 HA (Addgene plasmid # 8948, a gift from Philip Hinds) into the pBMN-IRES-Hygro vector (a gift from Gary Nolan) at XhoI and NotI restriction sites. CCND1-HA PCR products were generated using the following primer pairs: 5′ TAGTAGctcgagGCCG CCACCATGGAACACCAGCTCCTGTGC and 5′ CT ACTAGCGGCCGCTCAGATATCGGCGTAGTC. E36K, Y44D and C47S *CCND1* mutations were generated by the Quickchange Site-directed mutagenesis kit (Stratagene, La Jolla, CA) using the plasmid template pBMN-CCND1-HA-IRES-Hygro and primers as follows. E36K forward: 5′ P-ATGCTGAAGGCGGAGAAGACC TGCGCGCCCT, E36K reverse: 5′ P-GGCCCGCAGCAC CCGGTCGT, Y44D forward: 5′ P-GCGCCCTCGGTGT CCGACTTCAAATGTGTGC, Y44D reverse: 5′ P-GCA GGTCTCCTCCGCCTTCAGCAT, C47S forward: 5′ P-G TGTCCTACTTCAAATCTGTGCAGAAGGAGGT, C47S reverse: 5′ P-CGAGGGCGCGCAGGTCTCCTCCG. Mutations were confirmed by DNA sequencing. T286 *CCND1* mutation was derived from the plasmid pcDNA cyclin D1 HA T286A (Addgene plasmid #11182, a gift from Bruce Zetter) and subcloned into the pBMN-IRES-Hygro vector as described above.

### DNA transfection and retroviral transduction

DNA transfection was performed by mixing DNA with Lipofectamine2000 (Life Technologies, Grand Island, NY) and following the manufacturer's instructions. For retroviral transduction, a retroviral vector and a mixture of helper plasmids for viral envelope and *gag*/*pol* were transfected into HEK293T cells using Lipofectamine 2000. Retroviral supernatants were harvested 48 hours after transfection and were used to transduce ecotropic receptor-expressing target cells by centrifugation at 1200 × g for one hour in 4 μg/ml polybrene.

### Quantitative real-time PCR

SYBR^®^ Green-based quantitative real-time PCR was performed using RT^2^ SYBRÒ Green qPCR Mastermix (Qiagen, Valencia, CA) and a StepOnePlus Real-time PCR system (Life Technologies, Grand Island, NY). The following probes were used: CCND1 forward primer: 5′ CGAGGAGGAGGAAGAGGAG. HA reverse primer: 5′ GTAGTCCGGGACGTCGTA. GAPDH forward primer: 5′ AAGGGCTCATGACCACAGTC. GAPDH reverse primer: 5′ GGATGACCTTGCCCACAG. Relative mRNA expression was normalized to GAPDH signals and calculated using the ddCt method. Proximal and distal CCND1 UTR primers have been described previously [10]. BTK gene knockdown experiments using a previously validated shRNA sequence [42] (GCACAAACTCTCCTACTATGA) were analyzed by the StepOnePlus Real-time PCR system using the Applied Biosystems probe BTK (Hs00975865_m1) and GAPDH (Hs02758991_g1).

### Immunoblot and immunoprecipitation analyses

Cells were lysed in the presence of protease inhibitor cocktail (Sigma, St. Louis, MO) and Halt phosphatase inhibitor cocktail (Pierce Biotechnology, Rockford, IL) for 30 min. Lysates were cleared by centrifugation and protein concentrations were determined by BCA protein assay (Pierce Biotechnology, Rockford, IL). Ten or 30 μg of lysates per lane were separated by 4–15% SDS-PAGE and immobilized on the nitrocellulose membranes (ThermoFisher, Waltham, MA) for immunoblotting. Immunoblot signals were developed by a chemiluminescent detection method (Pierce Biotechnology, Rockford, IL) and captured by standard autoradiographic films. Signal intensities were quantitated using the NIH Image J software (imagej.nih.gov).

For immunoprecipitation, cells were lysed at 4 × 10^7^ cells/ml in IP buffer from Pierce Biotechnology (25 mM Tris·HCl pH 7.4, 150 mM NaCl, 1% NP-40, 1 mM EDTA, 5% glycerol), in presence of 1 mM PMSF, 10 mM glycerophosphate, 1× concentration of Protease and Halt Phosphatase cocktail inhibitors for 30 min on ice. Lysates were cleared by centrifuging for 15 minutes at 14,000 × g at 4°C. Five μg of IgG1 isotype control was mixed with 50 μl of 1:1 slurry of PBS and protein A agarose beads and the mixture was added to 1 ml of lysate followed by one-hour incubation on a rotating mixer at 4°C. Lysates were cleared again by centrifugation for one minute at 2400 × g at 4°C. Supernatants were quantified for protein concentrations using the BCA protein assay. Approximately, 1.5 mg of lysate from each sample was incubated with 40 μl of 1:1 slurry of PBS and HA antibody (IgG1)-conjugated agarose beads (Sigma, St. Louis, MO) overnight on a rotating mixer at 4°C. Agarose beads were washed 4 times in 1 ml of PBS containing 0.5% NP40 for 10 min each and pelleted by centrifugation at 2400 × g for 5 min. After discarding the supernatant, washed agarose beads were suspended in 100 μl of 1× sample buffer containing 5 μl of beta-mercaptoethanol and heat denatured for 5 min at 95°C. Samples were separated on 10% polyacrylamide gels and transferred to nitrocellulose membranes for western blot analysis.

For IP of Ub-conjugated CCND1, cells were harvested, washed twice with cold PBS, and lysed in 2% sodium dodecyl sulfate (SDS), 150 mM NaCl, 10 mM Tris-HCl pH 8.0, and protease inhibitors at 95°C for 5 min then kept on ice for 10 min, as previously described [43]. The lysate was then diluted in IP buffer containing protease and phosphatase inhibitors at ratio 1:10 and incubated on ice for 30 min. Lysates were cleared by centrifuging for 15 min at 14,000 × g at 4°C and used for immunoprecipitation as described above.

### Cell fractionation

Nine million cells were harvested and divided into two parts: 1 million cells were used for whole cell lysate and 8 million cells were used for fractionation. Cells were washed twice in PBS and treated with 250 μl of the Harvest buffer (10 mM HEPES pH 7.9, 50 mM NaCI, 0.5 M sucrose, 0.1 mM EDTA, 0.5% Triton X-100, with freshly added 1 mM DTT, protease and phosphatase inhibitors) for 5 min on ice. Lysates were cleared by centrifugation at 720 × g for 10 min in swinging bucket rotor at 4°C. Supernatants were transferred to new tubes, cleared by centrifugation at 14,000 × g for 15 min at 4°C and used as cytoplasmic fractions. The pellets from Harvest Buffer treatment were washed 2 to 3 times in 500 μl of Buffer A (10 mM HEPES pH 7.9, 10 mM KCI, 0.1 mM EDTA, 0.1 mM EGTA, and freshly added 1 mM DTT, protease and phosphatase inhibitors). Final pellets were lysed for 15 min at 4°C in Buffer B (10 mM HEPES pH 7.9, 500 mM NaCI, 0.1 mM EDTA, 0.1 mM EGTA, 0.1% NP-40, and freshly added 1 mM DTT, protease and phosphatase inhibitors). Pellets were sonicated and centrifuged for 15 min at 14,000 × g. Supernatants were used as nuclear fractions.

### Immunofluorescence

HEK-293T cells expressing WT or mutant CCND1-HA proteins were seeded at a density of 0.5 × 10^6^ cells/ml on a glass bottom 35 mm dish for 2 days. Cells were washed with PBS twice and fixed with 4% paraformaldehyde for 30 min at RT. Fixed cells were blocked with 5% BSA and 0.5% TritonX-100 in phosphate buffered saline (PBS) for 30 min at RT. Cells were then stained with anti-HA antibody for overnight at 4°C in a humidified chamber, followed by staining with fluorescently labeled secondary antibodies and nuclear staining with 4′,6-diamidino-2-phenylindole (DAPI) (Life Technologies, Grand Island, NY). Cells were observed using a confocal microscope (Inverted LSM510 Meta 2-Photon Microscope, Zeiss) with a 63X objective. Images were captured and analyzed using Zen Imaging Software (ZEISS USA, Dublin CA).

### Viability and apoptosis measurements

Cell viability was assessed either by using the CellTiter-Glo Luminescent assay (Promega, Madison, WI) following instructions by the manufacturer or by flow cytometric analysis for propidium iodide negative population. Apoptosis was measured by Annexin V-based methods using Annexin V Apoptosis Detection Kit (eBioscience, Inc., San Diego, CA).

### Sanger sequencing

Analysis of *CCND1* exon 1 by Sanger sequencing was performed using the CCND1 primers and method as described [23].

## SUPPLEMENTARY MATERIALS FIGURES



## References

[1] Matsushime H, Roussel MF, Ashmun RA, Sherr CJ (1991). Colony-stimulating factor 1 regulates novel cyclins during the G1 phase of the cell cycle. Cell.

[2] Kato J, Matsushime H, Hiebert SW, Ewen ME, Sherr CJ (1993). Direct binding of cyclin D to the retinoblastoma gene product (pRb) and pRb phosphorylation by the cyclin D-dependent kinase CDK4. Genes Dev.

[3] Lukas J, Pagano M, Staskova Z, Draetta G, Bartek J (1994). Cyclin D1 protein oscillates and is essential for cell cycle progression in human tumour cell lines. Oncogene.

[4] Diehl JA, Cheng M, Roussel MF, Sherr CJ (1998). Glycogen synthase kinase-3beta regulates cyclin D1 proteolysis and subcellular localization. Genes Dev.

[5] Diehl JA, Zindy F, Sherr CJ (1997). Inhibition of cyclin D1 phosphorylation on threonine-286 prevents its rapid degradation via the ubiquitin-proteasome pathway. Genes Dev.

[6] Zou Y, Ewton DZ, Deng X, Mercer SE, Friedman E (2004). Mirk/dyrk1B kinase destabilizes cyclin D1 by phosphorylation at threonine 288. J Biol Chem.

[7] Casanovas O, Miro F, Estanyol JM, Itarte E, Agell N, Bachs O (2000). Osmotic stress regulates the stability of cyclin D1 in a p38SAPK2-dependent manner. J Biol Chem.

[8] Williams ME, Meeker TC, Swerdlow SH (1991). Rearrangement of the chromosome 11 bcl-1 locus in centrocytic lymphoma: analysis with multiple breakpoint probes. Blood.

[9] Raffeld M, Jaffe ES (1991). bcl-1, t(11;14), and mantle cell-derived lymphomas. Blood.

[10] Wiestner A, Tehrani M, Chiorazzi M, Wright G, Gibellini F, Nakayama K, Liu H, Rosenwald A, Muller-Hermelink HK, Ott G, Chan WC, Greiner TC, Weisenburger DD (2007). Point mutations and genomic deletions in CCND1 create stable truncated cyclin D1 mRNAs that are associated with increased proliferation rate and shorter survival. Blood.

[11] Deshpande A, Pastore A, Deshpande AJ, Zimmermann Y, Hutter G, Weinkauf M, Buske C, Hiddemann W, Dreyling M (2009). 3′UTR mediated regulation of the cyclin D1 proto-oncogene. Cell Cycle.

[12] Lu F, Gladden AB, Diehl JA (2003). An alternatively spliced cyclin D1 isoform, cyclin D1b, is a nuclear oncogene. Cancer Res.

[13] Gladden AB, Woolery R, Aggarwal P, Wasik MA, Diehl JA (2006). Expression of constitutively nuclear cyclin D1 in murine lymphocytes induces B-cell lymphoma. Oncogene.

[14] Dal Col J, Zancai P, Terrin L, Guidoboni M, Ponzoni M, Pavan A, Spina M, Bergamin S, Rizzo S, Tirelli U, De Rossi A, Doglioni C, Dolcetti R (2008). Distinct functional significance of Akt and mTOR constitutive activation in mantle cell lymphoma. Blood.

[15] Jares P, Colomer D, Campo E (2007). Genetic and molecular pathogenesis of mantle cell lymphoma: perspectives for new targeted therapeutics. Nat Rev Cancer.

[16] Perez-Galan P, Dreyling M, Wiestner A (2011). Mantle cell lymphoma: biology, pathogenesis, and the molecular basis of treatment in the genomic era. Blood.

[17] Wang ML, Rule S, Martin P, Goy A, Auer R, Kahl BS, Jurczak W, Advani RH, Romaguera JE, Williams ME, Barrientos JC, Chmielowska E, Radford J (2013). Targeting BTK with ibrutinib in relapsed or refractory mantle-cell lymphoma. N Engl J Med.

[18] Chiron D, Di Liberto M, Martin P, Huang X, Sharman J, Blecua P, Mathew S, Vijay P, Eng K, Ali S, Johnson A, Chang B, Ely S (2014). Cell-cycle reprogramming for PI3K inhibition overrides a relapse-specific C481S BTK mutation revealed by longitudinal functional genomics in mantle cell lymphoma. Cancer discovery.

[19] Rahal R, Frick M, Romero R, Korn JM, Kridel R, Chan FC, Meissner B, Bhang HE, Ruddy D, Kauffmann A, Farsidjani A, Derti A, Rakiec D (2014). Pharmacological and genomic profiling identifies NF-kappaB-targeted treatment strategies for mantle cell lymphoma. Nature medicine.

[20] Ma J, Lu P, Guo A, Cheng S, Zong H, Martin P, Coleman M, Wang YL (2014). Characterization of ibrutinib-sensitive and -resistant mantle lymphoma cells. Br J Haematol.

[21] Furman RR, Cheng S, Lu P, Setty M, Perez AR, Guo A, Racchumi J, Xu G, Wu H, Ma J, Steggerda SM, Coleman M, Leslie C (2014). Ibrutinib resistance in chronic lymphocytic leukemia. N Engl J Med.

[22] Woyach JA, Furman RR, Liu TM, Ozer HG, Zapatka M, Ruppert AS, Xue L, Li DH, Steggerda SM, Versele M, Dave SS, Zhang J, Yilmaz AS (2014). Resistance mechanisms for the Bruton's tyrosine kinase inhibitor ibrutinib. N Engl J Med.

[23] Kridel R, Meissner B, Rogic S, Boyle M, Telenius A, Woolcock B, Gunawardana J, Jenkins C, Cochrane C, Ben-Neriah S, Tan K, Morin RD, Opat S (2012). Whole transcriptome sequencing reveals recurrent NOTCH1 mutations in mantle cell lymphoma. Blood.

[24] Bea S, Valdes-Mas R, Navarro A, Salaverria I, Martin-Garcia D, Jares P, Gine E, Pinyol M, Royo C, Nadeu F, Conde L, Juan M, Clot G (2013). Landscape of somatic mutations and clonal evolution in mantle cell lymphoma. Proc Natl Acad Sci U S A.

[25] Zhang J, Jima D, Moffitt AB, Liu Q, Czader M, Hsi ED, Fedoriw Y, Dunphy CH, Richards KL, Gill JI, Sun Z, Love C, Scotland P (2014). The genomic landscape of mantle cell lymphoma is related to the epigenetically determined chromatin state of normal B cells. Blood.

[26] Weinstein S, Emmanuel R, Jacobi AM, Abraham A, Behlke MA, Sprague AG, Novobrantseva TI, Nagler A, Peer D (2012). RNA inhibition highlights cyclin D1 as a potential therapeutic target for mantle cell lymphoma. PLoS One.

[27] Mohanty S, Mohanty A, Sandoval N, Tran T, Bedell V, Wu J, Scuto A, Murata-Collins J, Weisenburger DD, Ngo VN (2016). Cyclin D1 depletion induces DNA damage in mantle cell lymphoma lines. Leuk Lymphoma.

[28] Sutherland C, Leighton IA, Cohen P (1993). Inactivation of glycogen synthase kinase-3 beta by phosphorylation: new kinase connections in insulin and growth-factor signalling. The Biochemical journal.

[29] Stambolic V, Woodgett JR (1994). Mitogen inactivation of glycogen synthase kinase-3 beta in intact cells via serine 9 phosphorylation. The Biochemical journal.

[30] Cross DA, Alessi DR, Cohen P, Andjelkovich M, Hemmings BA (1995). Inhibition of glycogen synthase kinase-3 by insulin mediated by protein kinase B. Nature.

[31] Coghlan MP, Culbert AA, Cross DA, Corcoran SL, Yates JW, Pearce NJ, Rausch OL, Murphy GJ, Carter PS, Roxbee Cox L, Mills D, Brown MJ, Haigh D (2000). Selective small molecule inhibitors of glycogen synthase kinase-3 modulate glycogen metabolism and gene transcription. Chemistry & biology.

[32] Stambolic V, Ruel L, Woodgett JR (1996). Lithium inhibits glycogen synthase kinase-3 activity and mimics wingless signalling in intact cells. Current biology.

[33] Lannutti BJ, Meadows SA, Herman SE, Kashishian A, Steiner B, Johnson AJ, Byrd JC, Tyner JW, Loriaux MM, Deininger M, Druker BJ, Puri KD, Ulrich RG (2011). CAL-101, a p110delta selective phosphatidylinositol-3-kinase inhibitor for the treatment of B-cell malignancies, inhibits PI3K signaling and cellular viability. Blood.

[34] Zeng Z, Shi YX, Tsao T, Qiu Y, Kornblau SM, Baggerly KA, Liu W, Jessen K, Liu Y, Kantarjian H, Rommel C, Fruman DA, Andreeff M (2012). Targeting of mTORC1/2 by the mTOR kinase inhibitor PP242 induces apoptosis in AML cells under conditions mimicking the bone marrow microenvironment. Blood.

[35] Bodrug SE, Warner BJ, Bath ML, Lindeman GJ, Harris AW, Adams JM (1994). Cyclin D1 transgene impedes lymphocyte maturation and collaborates in lymphomagenesis with the myc gene. EMBO J.

[36] Lovec H, Grzeschiczek A, Kowalski MB, Moroy T (1994). Cyclin D1/bcl-1 cooperates with myc genes in the generation of B-cell lymphoma in transgenic mice. EMBO J.

[37] Katz SG, Labelle JL, Meng H, Valeriano RP, Fisher JK, Sun H, Rodig SJ, Kleinstein SH, Walensky LD (2014). Mantle cell lymphoma in cyclin D1 transgenic mice with Bim-deficient B cells. Blood.

[38] Yamamoto K, Lee BJ, Li C, Dubois RL, Hobeika E, Bhagat G, Zha S (2015). Early B-cell-specific inactivation of ATM synergizes with ectopic CyclinD1 expression to promote pre-germinal center B-cell lymphomas in mice. Leukemia.

[39] Alt JR, Cleveland JL, Hannink M, Diehl JA (2000). Phosphorylation-dependent regulation of cyclin D1 nuclear export and cyclin D1-dependent cellular transformation. Genes Dev.

[40] Martin P, Maddocks K, Leonard JP, Ruan J, Goy A, Wagner-Johnston N, Rule S, Advani R, Iberri D, Phillips T, Spurgeon S, Kozin E, Noto K (2016). Postibrutinib outcomes in patients with mantle cell lymphoma. Blood.

[41] M'Kacher R, Farace F, Bennaceur-Griscelli A, Violot D, Clausse B, Dossou J, Valent A, Parmentier C, Ribrag V, Bosq J, Carde P, Turhan AG, Bernheim A (2003). Blastoid mantle cell lymphoma: evidence for nonrandom cytogenetic abnormalities additional to t(11;14) and generation of a mouse model. Cancer genetics and cytogenetics.

[42] Davis RE, Ngo VN, Lenz G, Tolar P, Young RM, Romesser PB, Kohlhammer H, Lamy L, Zhao H, Yang Y, Xu W, Shaffer AL, Wright G (2010). Chronic active B-cell-receptor signalling in diffuse large B-cell lymphoma. Nature.

[43] Choo YS, Zhang Z (2009). Detection of protein ubiquitination. Journal of visualized experiments.

