# Supplementary material for: CCND1 mutations increase protein stability and promote ibrutinib resistance in mantle cell lymphoma
Source: Oncotarget. 2016 Oct 4;7(45):73558–72. doi: 10.18632/oncotarget.12434 (PMC5341999; doi:10.18632/oncotarget.12434)
Supplement: Supplementary file 1 [file oncotarget-07-73558-s001.pdf]

CCND1 mutations increase protein stability and promote ibrutinib resistance in mantle cell lymphoma

Supplementary Materials

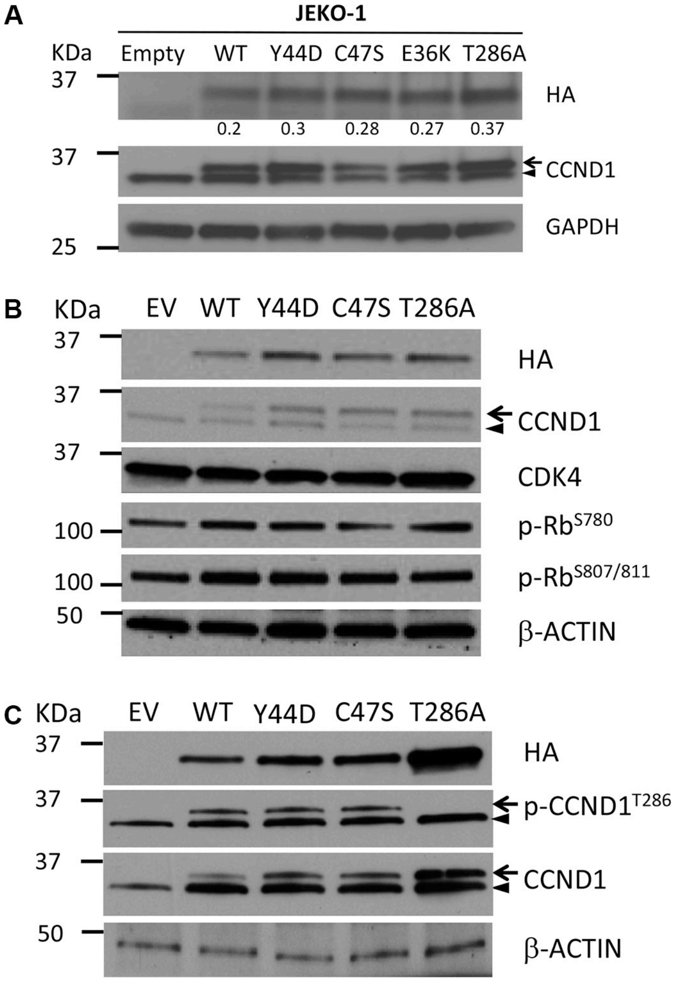

Supplementary Figure S1: Effects of *CCND1* mutations on protein levels CDK4 activity and T286 phosphorylation. (**A**–**C**) JEKO-1 cells were transduced with WT or mutant CCND1-HA and selected for stable expression by hygromycin. Cell lysates (10 µg per lane) were separated by SDS-PAGE gel and immunoblotted with indicated antibodies. Arrow indicates a mobility shift of the CCND1-HA protein. Arrowhead indicates endogenous CCND1. Numbers below the blot in A are relative densitometric values of corresponding bands after normalization to GAPDH loading controls. (B) The catalytic activity of CDK4 in cells that express empty vector (EV), WT or mutant CCND1 was analyzed by phosphorylation of Rb, a substrate of CDK4. (C) Phosphorylation of T286 was analyzed in cells that expressed WT or mutant CCND1. The T286A mutant serves as a negative control.

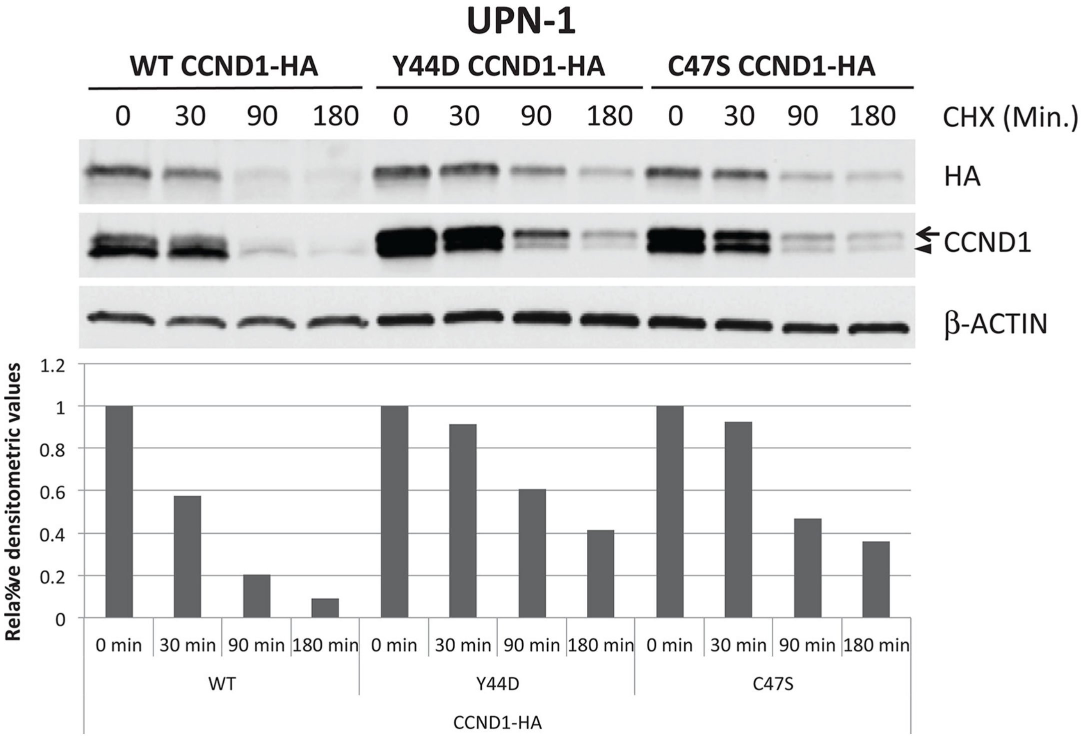

Supplementary Figure S2: CCND1 mutations affect protein turnover. UPN-1 cells expressing WT, Y44D or C47S CCND1-HA were treated with 10 μM of cyclohexamide (CHX) for the indicated times and 10 μg of cell lysate per lane was prepared for immunoblot analysis with the indicated antibodies. Arrow, CCND1-HA protein. Arrowhead, endogenous CCND1. Bar graphs below the blot show relative densitometric values of CCND1-HA bands from the blot after normalization to time zero control samples.

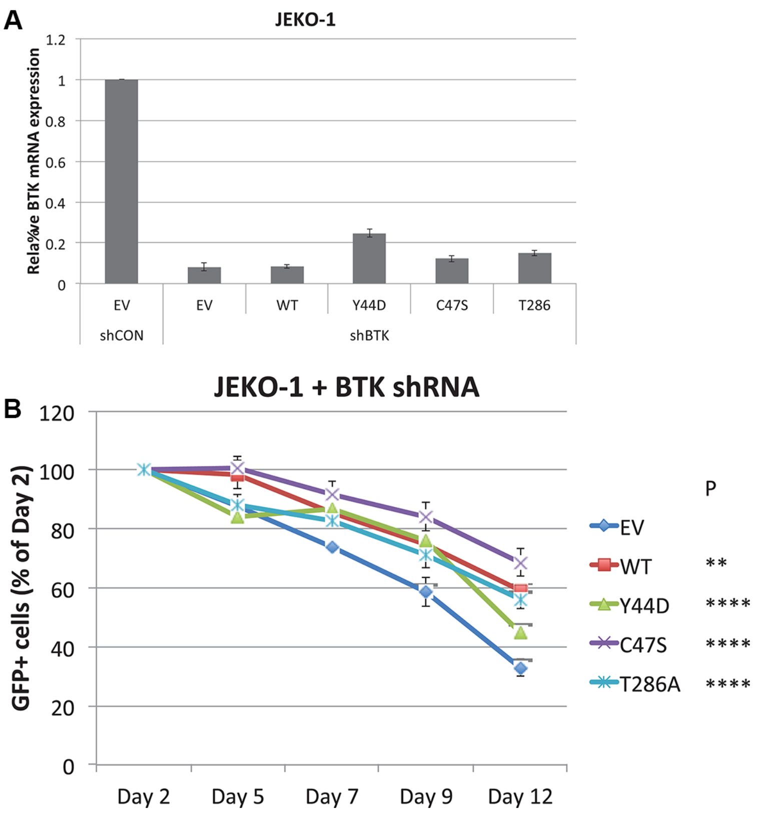

Supplementary Figure S3: CCND1 mutations rescue BTK shRNA toxicity. JEKO-1 cells expressing an empty vector (EV), WT, or indicated mutant CCND1-HA were transduced with a retrovirus that co-expressed BTK shRNA and GFP. (**A**) Verification of BTK knockdown. Control and BTK shRNA-transduced JEKO-1 cells were selected by puromycin and analyzed for relative BTK mRNA expression by qPCR on day 3 after transduction. Shown are the means of 3 independent experiments. Error bars, SD. (**B**) Overexpression of WT or mutant CCND1 rescues cells from BTK shRNA toxicity. Shown are the normalized fractions (to day 2 values) of GFP^+, BTK shRNA-expressing cells relative to GFP^-, shRNA-negative fractions at indicated times, as assessed by flow cytometry. The drop in GFP+ fractions over time indicates BTK shRNA toxicity. Line graphs show the means of 3 independent experiments. Error bars, SD. ****$P < 0.0001$, **$P < 0.01$ (two-way ANOVA). *P* values indicate significance levels for the rescue effect of WT or mutants compared to empty vector (EV) control.

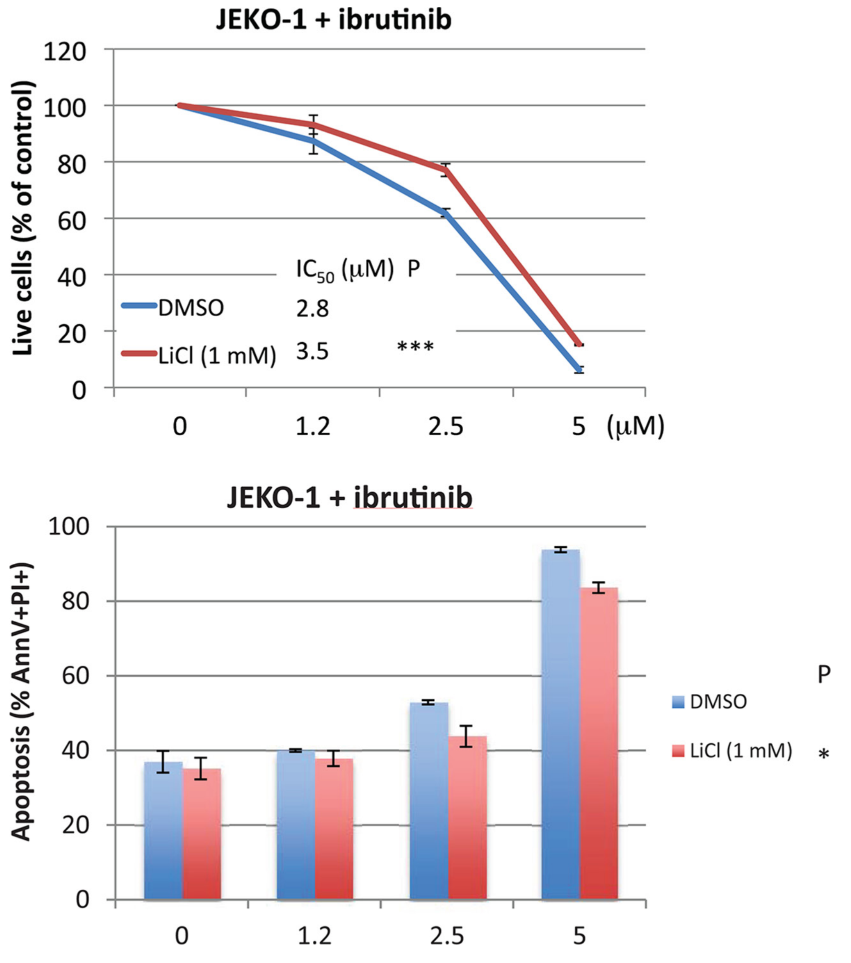

Supplementary Figure S4: Ibrutinib toxicity in JEKO-1 cells is GSK3B dependent. JEKO-1 cells were co-treated with indicated doses of ibrutinib and 1 mM of LiCl or DMSO control. Viable, propidium idodide (PI)-negative cells (top panel) or Annexin+ PI+ apoptotic cells (bottom panel) were assessed by flow cytometry on day 4. Shown in line graphs are PI negative fractions normalized to control untreated samples. Line graphs or bar graphs show means of three independent experiments. Error bars, SD. ***$P < 0.001$, *$P < 0.05$ (two-way ANOVA). *P* values indicate significance levels for the effect of LiCl, compared to DMSO, on ibrutinib sensitivity.
